# LC-Q-TOF-MS/MS data of phyllobilins in apple peels cv. ‘Gala’ during ripening under shelf-life conditions

**DOI:** 10.1016/j.dib.2023.109259

**Published:** 2023-05-23

**Authors:** Lisa Marie Gorfer, Luca Vestrucci, Valentina Grigoletto, Valentina Lazazzara, Angelo Zanella, Peter Robatscher, Matteo Scampicchio, Michael Oberhuber

**Affiliations:** a Laimburg Research Centre, Laimburg 6 – Pfatten (Vadena), 39040 Auer (Ora), BZ, Italy; b Faculty of Agricultural, Environmental and Food Sciences, Free University of Bozen-Bolzano, Piazza Università 5, 39100 Bolzano, Italy

**Keywords:** High Resolution Mass Spectrometry, Chlorophyll breakdown, Fruit ripening, Inclusion list, Data dependent acquisition, Nonfluorescent Chlorophyll Catabolites, Apple (Malus×domestica Borkh.)

## Abstract

During senescence and ripening, higher plants degrade the green pigment chlorophyll to linear tetrapyrrols, referred to as phyllobilins (PBs). This dataset provides chromatograms and mass spectral data of PBs acquired from methanolic extracts of cv. Gala apple peels at five different shelf life (SL) stages. Data were obtained using a (ultra-high) pressure liquid chromatograph (UHPLC) coupled to high resolution quadrupole-time of flight-mass spectrometer (HRMS-Q-TOF). A data-dependent inclusion list (IL) *ex professo* containing all known masses of PBs was applied to address PBs, and fragmentation patterns were studied to confirm their identity operating a MS^2^ method. Mass accuracy was set to 5 ppm for parent ion peaks, this parameter was adopted as inclusion criterium. The detection of PBs’ appearance during ripening can be helpful for assessing the quality and maturity of the apples.

**Specifications Table**

**Table:** 

Subject	Agricultural Sciences
Specific subject area	Metabolomics, Natural Product Research, High Resolution Mass Spectrometry, Climacteric Fruits Ripening.
Type of data	Table Figure Chromatogram including Mass spectrum
How the data were acquired	Methanolic extracts of apple peels were analyzed. Mass spectral data were acquired using (ultra-high) pressure liquid chromatography (UHPLC) coupled to high resolution quadrupole-time of flight-mass spectrometry (Q-TOF-MS). A Dionex UltiMate 3000 UHPLC (Thermo Scientific, Darmstadt, Germany) coupled to an Impact HD Q-TOF-MS (Bruker Daltonics, Bremen, Germany) was employed. Data were analyzed with Compass DataAnalysis 4.2 software (Bruker Daltonics, Bremen, Germany).
Data format	Raw Analyzed
Description of data collection	Apples (*Malus* × *domestica* Borkh.) of cv. ‘Gala’ (clone ‘Schniga’) were collected in September 2018 from an experimental field in Laces (670 m a.s.l.), Val Venosta, South Tyrol, Italy, at their optimal harvest date with a starch index of 4.2, based on the CTIFL scale using the Starch Iodine Test. The apples were stored under shelf life conditions (room temperature and ambient daylight), and15 apples were sampled weekly for five weeks. Three of 15 apples of each time point were selected for phyllobilin extraction and analyzed with LC-Q-TOF-MS/MS.
Data source location	Solvent extraction, LC-Q-TOF-MS/MS (ESI^+^) analysis, and data acquisition and storage were performed at the Laboratory for Flavours and Metabolites of Laimburg Research Centre. The laboratory is located at NOI Techpark Südtirol/Alto Adige, 13 Alessandro Volta Street, 39100 Bolzano, Italy (46°28′43.7″N 11°19′52.7″E).
Data accessibility	Repository name: zenodo.org Digital Object Identifier (DOI): 10.5281/zenodo.7912642 Direct URL to data: https://doi.org/10.5281/zenodo.7912642
Related research article	L. M. Gorfer, L. Vestrucci, V. Grigoletto, V. Lazazzara, A. Zanella, P. Robatscher, M. Scampicchio, M. Oberhuber. Chlorophyll breakdown during fruit ripening: Qualitative analysis of phyllobilins in the peel of apples (*Malus domestica* Borkh.) cv. ‘Gala’ during different shelf life stages. Food Res. Int. (162), 2022, 112061. https://doi.org/10.1016/j.foodres.2022.112061.

## Value of the Data

• These data provide a comprehensive high-resolution mass-spectrometric (HR-MS) data analysis of PBs detected apple peels.
• These data contain chromatograms and mass spectra acquired from peel extracts of fruits at different ripening stages.
• In these data we present, for the first time, a literature-based, complete inclusion list containing all known (39) PBs, 25 hypothesized PBs, and detailed information such as molecular formula, constitutional formula, pseudo molecular ion and fragmentation patterns for HR-MS analysis.
• These data can be helpful for identification and structure elucidation of novel PBs in higher plants.
• These data can be applied for agronomical purpose to predict optimal harvest dates of apples.

## 1 Objective

Chlorophyll degradation occurs during fruit maturation, giving rise to structurally diverse linear tetrapyrrols, referred to as phyllobilins (PBs), via the pheophorbide a oxygenase (PaO)/PB pathway [1].

The dataset was generated to analyse the full spectrum PBs, deduced from all currently published plant sources, during fruit ripening. Apple was chosen as a climacteric model fruit. In the related article, we studied methanolic extracts of apple peels at different maturity states using liquid chromatography coupled to high-resolution quadrupole-time-of-flight-mass spectrometry (LC-HRMS-Q-TOF) [2]. Both the LC-Q-TOF-MS/MS method and the data-dependent inclusion list (IL) *ex professo* are useful to study chlorophyll breakdown products in other fruits and vegetables. Here, we publish a complete set of the chromatographic and mass-spectrometric data obtained from apple peel extracts to enable researchers to perform similar studies in a wide range of plant sources.

## 2 Data Description

(1) The data-dependent inclusion list (IL) *ex professo* (Table DR1 in Data Repository) contains all PBs employed to detect them in apple peel extracts. Names of PBs used in literature (column A), molecular formulae (column B), *m*/*z* (M+H)^+^ theoretical (column C), the chemical constitutions with the different side chain residues in R1 (column E), R2 (column F), R3 (column G) and R4 (column H). Additionally, typical fragments obtained in MS^2^ (column I), fragment formula (column J), neutral loss (column K) and *m/z* theoretical (column L) are listed.
(2) The figures show a generalized constitutional PB formula (Fig. DR1 in Data Repository) and the constitutional formula of a specific PB example (NCC-1002), including its characteristic fragmentation sides (Fig. DR2 in Data Repository).
(3) In Tables DR2 to DR16 in Data Repository the base peak chromatograms (BPCs) of all peaks detected at five different ripening stages (0, 7, 14, 21, 30 SL days) in the peel extracts of three apples (apple1, apple2, apple3 per ripening stage) are outlined. These tables include progressive number which corresponds to a peak number (column A), retention time (r.t.) (column B), peak area (column C), peak intensity (column D), signal-to-noise (S/N) ratio (column E) and full width at half maximum (FWHM) (column F).
(4) In Tables DR17 to DR31 in Data Repository the extracted ion chromatograms (EICs) of all PBs detected at five different ripening stages (0, 7, 14, 21, 30 SL days) in the peel extracts of three apples (apple1, apple2, apple3 per ripening stage) are outlined. These tables include progressive number which corresponds to a PB (column A); they provide nomenclature adopted in literature (column B), denomination in this work (column C), formula (column D), *m/z* (M+H)^+^ calculated (column E) and *m/z* (M+H)^+^ measured (column F), mass error (column G), and retention time (r.t.) (column H). We also added information regarding typical PB fragmentation in MS^2^ (column I), including fragment formula (column J), neutral loss (column K), *m/z* (fragment) calculated MS^2^ (column L), *m/z* (fragment) measured MS^2^ (column M) and the fragment mass error (column N).
(5) Bruker mass spectrometer files (Chromatograms BPC + All MS and EIC of PBs in Data Repository): all data are mass calibrated (internal) and contain BPC +All MS, TIC (Total Ion Chromatogram) +All MSn and EICs +All MS of the 10 PBs with a mass width set to ± 0.01 found in this study.

## 3 Experimental Design, Materials and Methods

### 3.1 Sample Collection and Preparation

Apples cv. ‘Gala’ were harvested from an experimental field in Laces (670 m a.s.l.), Val Venosta, South Tyrol, Italy on September 3^rd^, 2018. They were collected on the optimal harvest date estimated with a starch index of 4.2 value (CTFL starch conversion color chart for apples, [3]). Apples were stored at room temperature (20-25°C), ambient daylight and 60-70% relative humidity for 30 days to mimic ripening.

### 3.2 PBs Extraction and Analysis

Apples were sampled after 0, 7, 14, 21 and 30 SL days. Apple peels were removed with a kitchen peeler, weighted, and stored individually in centrifuge tubes at −80°C until analysis. A spatula of CaCO_3_ was added (ca. 10% of the peel weight); samples were frozen in liquid nitrogen and homogenized in a mixer mill Retsch MM400 for 30 s at 30 Hz. PBs extraction from peels was performed with methanol into two steps (7 + 3 mL). The combined extracts were centrifugated (12,000 rpm, 4°C, 10 min), a 5 mL aliquot of the supernatant was concentrated to dryness using a Speedvac, and the residue was reconstituted with 50 µL LC-MS eluent [methanol (solvent B): 4 mM aqueous ammonium acetate (solvent A), 1:1 (*v/v*)]. UHPLC-Q-TOF-MS analysis was conducted as described in Tables 1 and 2.

**Table 1:** LC gradient.

Time (min)	Flow rate (mL min^−1^)	Solvent A [4 mM aqueous ammonium acetate (*v/v*)]	Solvent B [methanol (*v/v*)]
0-5	0.5	80%	20%
5-55	0.5	30%	70%
55-60	0.5	5%	95%
60-70	0.5	5%	95%
70-75	0.5	80%	20%
75-85	0.5	80%	20%

**Table 2:** LC system and Q-TOF-MS/MS system parameters.

Column	Phenomenex HyperClone ODS C18
Column dimension	120 Å, 5 μm, 250 × 4.6 mm
Column temperature	25°C
Flow rate	0.50 mL min^−1^
UV Detection (DAD)	200 – 700 nm
Injection volume	20 µL
Source type	Electrospray ionization
Ion polarity	positive
Mass scan range	50-1500 *m/z*
Set capillary	4500 V
Set end plate offset	500 V
Set charging voltage	2000 V
Set nebulizer	3.0 bar
Set dry gas	12.0 L min^−1^
Set dry temperature	230°C

A total of 10 PBs were detected in all samples under investigation: five so-called nonfluorescent chlorophyll catabolites (NCCs), one yellow chlorophyll catabolite (YCC) and four nonfluorescent dioxobilin-type chlorophyll catabolites (DNCCs).

## Ethics Statements

This work does not involve any type of human studies, animal studies, or data gathered using social media.

## Funding

This research was funded by the Laimburg Research Centre, Project *‘Monitoring chlorophyll and its breakdown products as non-destructive tool for the precise prediction of apple postharvest quality (MoChAp)’* (Outside the box - project nr. LCH-am-19-5).

## CRediT authorship contribution statement

**Lisa Marie Gorfer:** Data curation, Formal analysis, Investigation, Methodology, Software, Validation, Visualization, Writing – original draft. **Luca Vestrucci:** Data curation, Formal analysis, Investigation, Software, Validation, Visualization, Writing – original draft. **Valentina Grigoletto:** Data curation, Investigation, Methodology, Software, Writing – review & editing. **Valentina Lazazzara:** Conceptualization, Funding acquisition, Writing – review & editing. **Angelo Zanella:** Conceptualization, Funding acquisition, Investigation, Writing – review & editing. **Peter Robatscher:** Conceptualization, Data curation, Funding acquisition, Investigation, Project administration, Resources, Supervision, Validation, Writing – review & editing. **Matteo Scampicchio:** Writing – review & editing. **Michael Oberhuber:** Data curation, Resources, Supervision, Validation, Writing – review & editing.

## Declaration of Competing Interest

The authors declare that they have no known competing financial interests or personal relationships that could have appeared to influence the work reported in this paper.

## Data Availability

Chromatographic and spectroscopic data (LC-MS) (Original data) (Zenodo).
